# Systems Biology Strategy Reveals PKCδ is Key for Sensitizing TRAIL-Resistant Human Fibrosarcoma

**DOI:** 10.3389/fimmu.2014.00659

**Published:** 2015-01-05

**Authors:** Kentaro Hayashi, Sho Tabata, Vincent Piras, Masaru Tomita, Kumar Selvarajoo

**Affiliations:** ^1^Institute for Advanced Biosciences, Keio University, Tsuruoka, Japan; ^2^Systems Biology Program, Graduate School of Media and Governance, Keio University, Fujisawa, Japan

**Keywords:** TRAIL, protein kinase C, signaling pathway, cancer, apoptosis, cell dynamics, computational model

## Abstract

Cancer cells are highly variable and largely resistant to therapeutic intervention. Recently, the use of the tumor necrosis factor related apoptosis-inducing ligand (TRAIL) induced treatment is gaining momentum due to TRAIL’s ability to specifically target cancers with limited effect on normal cells. Nevertheless, several malignant cancer types still remain non-sensitive to TRAIL. Previously, we developed a dynamic computational model, based on perturbation-response differential equations approach, and predicted protein kinase C (PKC) as the most effective target, with over 95% capacity to kill human fibrosarcoma (HT1080) in TRAIL stimulation (1). Here, to validate the model prediction, which has significant implications for cancer treatment, we conducted experiments on two TRAIL-resistant cancer cell lines (HT1080 and HT29). Using PKC inhibitor bisindolylmaleimide I, we demonstrated that cell viability is significantly impaired with over 95% death of both cancer types, in consistency with our previous model. Next, we measured caspase-3, Poly (ADP-ribose) polymerase (PARP), p38, and JNK activations in HT1080, and confirmed cell death occurs through apoptosis with significant increment in caspase-3 and PARP activations. Finally, to identify a crucial PKC isoform, from 10 known members, we analyzed each isoform mRNA expressions in HT1080 cells and shortlisted the highest 4 for further siRNA knock-down (KD) experiments. From these KDs, PKCδ produced the most cancer cell death in conjunction with TRAIL. Overall, our approach combining model predictions with experimental validation holds promise for systems biology based cancer therapy.

## Introduction

Numerous recent studies have revealed the close link between inflammation and cancer. First, various types of immune cells, which support tumor growth progression, are found within the tumor microenvironment (2, 3). Second, the vicinity of cancer cells displays increased proinflammatory activity, through the detection of elevated levels of major cytokines such as the tumor necrosis factor (TNF) (4, 5). One notable cytokine found within the tumor microenvironment is the TNF related apoptosis-inducing ligand or TRAIL, which has been shown to induce apoptosis in certain types of malignant cancers with no significant effect on normal cells (6, 7). The findings have led to a major stride in the ongoing research aimed at optimizing TRAIL-induced cancer therapy (8, 9). Despite some success, TRAIL-based therapies still show dismal results for several types of cancers such as the breast cancer, neuroblastoma, adenocarcinoma, and glioma (10–13).

Computational modeling approaches are becoming increasing useful for interpreting complex dynamical cellular responses (14–21). Previously, to understand the mechanism for TRAIL-resistance in cancer, we developed a dynamic computational model of TRAIL signaling, from extracellular receptor activation to downstream intracellular activation of cell survival (MAP kinases and IκB) and apoptosis (caspases-8 and -3) pathways (1). Our model was based on perturbation-response approach utilizing first-order response equations (1, 22–29), which was shown to successfully simulate the temporal experimental profiles IκB, JNK, p38, caspase-8 and -3 in wildtype, and four (FADD, RIP1, TRAF2, and caspase-8) knock-down (KD) conditions for human fibrosarcoma (30). We, subsequently, predicted targeting a novel molecule interacting with p62 in the model would significantly increase caspase-3 activation and enhance cancer apoptosis to TRAIL stimulation. Further protein-protein interaction (PPI) database analysis suggested that the novel molecule is most probably a protein kinase C (PKC) family member.

Here, we tested the model prediction by experimentally verifying whether targeting PKC will enhance apoptosis in TRAIL-resistant cancer cell lines. Experiments were performed on TRAIL-induced human fibrosarcoma (HT1080) and human colon adenocarcinoma (HT29) cells, and the cell viability was compared with control normal fibroblasts (TIG-1 and MRC-5). Moreover, to investigate the intracellular mechanisms for resultant cell viability, we measured time-course activation levels of caspase-3, PARP, p38, and JNK. Subsequently, we analyzed the expressions of each PKC isoform member in HT1080 cells. To identify a crucial target member for enhanced cancer apoptosis, we prepared relevant siRNA KD experiments. In summary, our study investigates (i) whether the model prediction of PKC suppression will enhance cancer cell death is true, and (ii) whether computational modeling using perturbation-response approach is valuable for biological research focusing on cancer treatment.

## Materials and Methods

### Reagents and cell culture

Recombinant human TRAIL was purchased from Peprotech. Bisindolylmaleimide I (BIM-I) was purchased from Merck Millipore. Human fibrosarcoma cell lines (HT1080), human embryo fibroblasts (TIG-1), and human colorectal adenocarcinoma cells (HT29) were obtained from Japanese Collection of Research Bioresources (JCRB) cell bank. Human fetal lung fibroblasts (MRC-5) were obtained from American Type Culture Collection (ATCC). HT1080, TIG-1, HT29, and MRC-5 were grown in DMEM (Nissui Seiyaku Co.) containing 10% calf serum, 100 U/mL of penicillin, Streptomycin 100 μg/ml and Amphotericin B 0.25 μg/ml at 37°C in a 5% CO_2_ humidified atmosphere.

### Cell viability assay

The cell viability was measured by 3-(4,5-dimethylthiazol-2-yl)-2,5-diphenyltetrazolium bromide (MTT) assay and trypan blue exclusion. MTT assay: cells (10 × 10^4^) were inoculated in each well and incubated for 24 h. Thereafter, 50 μL of MTT (2 mg/mL in PBS) was added to each well and the plates were incubated for a further 2 h. The resultant formazan was dissolved with 100 μL of dimethyl sulfoxide (DMSO) after aspiration of culture medium. Plates were placed on a plate shaker for 1 min and then read immediately at 570 nm using TECAN microplate reader with Magellan software (Männedorf, Switzerland). Trypan blue exclusion: cells were detached with 1 mL of trypsin and suspended in DMEM. After staining with trypan blue, viable cells were counted using microscopy (*n* = 3). The percentage of trypan blue exclusive viable cells was determined as a percentage of the total number of cells.

### Western blot analysis

Anti-PARP, anti-phospho-p38, and anti-β-actin antibodies were purchased from Cell Signaling Technology. Proteins were extracted from the cell lines using radioimmunoprecipitation assay (RIPA) buffer according to the manufacturer’s instructions. Next, their concentrations were measured by Bradford protein assay. Equal amounts of protein were loaded in each well and separated by 10% sodium dodecyl sulfate-polyacrylamide gel electrophoresis (SDS-PAGE), which was subsequently transferred onto a polyvinylidene difluoride (PVDF) membrane. The membrane was blocked for 1 h with 5% BSA in TBST on the shaker at room temperature. The membrane was placed on PARP and p–p38 antibody diluted at a 1:1000 proportion in diluent buffer [5% (w/v) BSA and 0.1% Tween 20 in TBS] and incubated overnight at 4°C on the shaker. The membrane was washed three times in TBS as above and incubated with secondary antibody diluted at a 1:10000 proportion for 1 h on the shaker at room temperature. The membrane was again washed three times for 5 min each time as above and finally the results were generated by using an enhanced chemiluminescence (ECL) Western blotting kit.

### Enzyme linked immunosorbent assays of cleaved caspase-3 and phosphorylated JNK

Cleaved caspase-3 and phosphorylated JNK concentrations were measured by ELISA Duo Sets IC Kit (R&D Systems) following the instructions of the manufacturer.

### Transfection

siRNA duplexes were purchased from Sigma. The transfection of classic PKCs (PKCα, PKCβ, PKCγ), the novel PKCs (PKCδ, PKCε, PKCη, PKCμ, PKCθ), and the atypical PKCs (PKCζ, PKCι) and scrambled siRNA were carried out using Lipofectamine 2000 according to the manufacturer’s instructions (Invitrogen).

### Quantitative real-time PCR analysis

Total cellular RNA was extracted from cells using the TRIzol reagent according to the manufacturer’s instructions (Invitrogen). One microgram of RNA was reverse-transcribed using a first-strand cDNA synthesis kit (ReverTra Aceα; Toyobo). Quantitative real-time PCR (qRT-PCR) was performed using SYBR premix Ex Taq (Takara) on the Applied Biosystems StepOnePlus™ according to the technical brochure of the company. qRT-PCR primers used in this study are listed in Table 1. Quantitative measurements were determined using the ΔΔCt method and expressions of GAPDH gene for PKC genes and RPL27 gene for *rela*, *mtor*, *bcl2*, *bax*, *cytoc*, and *jun* were used as the internal control. Melt curve analyses of all qRT-PCR products were performed and shown to produce the sole DNA duplex.

**Table 1:** **List of primer sequences for qRT-PCR**.

Name	Species	Primer name	Sequence(5′–3′)
*PKC*α	Human	PKCα_F	CCACACTAAATCCGCAGTGG
	Human	PKCα_R	CAGCTCCGAAACTCCAAAGGA
*PKC*β	Human	PKCβ_F	TTGTGGACCTGAAGGCGAAC
	Human	PKCβ_R	CGGGTGAAAAATCGGTCGAAG
*PKC*γ	Human	PKCγ_F	GCTTGTAACTACCCCCTGGAAT
	Human	PKCγ_R	GAAGCTGAAGTCGGAGATGTG
*PKC*δ	Human	PKCδ_F	TGGTGGTTGGTGCGTTGTAG
	Human	PKCδ_R	ATAGGAGTTGAAGGCGATGCG
*PKC*ε	Human	PKCε_F	CAAGCCACCCTTCAAACCAC
	Human	PKCε_R	CGTCCACAAGGGTGAGTACC
*PKC*η	Human	PKCη_F	GTGTCGTCCATAAACGCTGC
	Human	PKCη_R	ATCCCGAACCTCTGTTCTGC
*PKC*μ	Human	PKCμ_F	GAGGACGCCAACAGAACCAT
	Human	PKCμ_R	CCTTGCTGGTGTAGTGGACC
*PKC*θ	Human	PKCθ_F	GCTGATTGGTCAGTCGCCTT
	Human	PKCθ_R	TCTTCTCAGGTTCTCGCACG
*PKC*ζ	Human	PKCζ_F	CACATGCAGAGGCAGAGGAA
	Human	PKCζ_R	GAGGACGTTGTCCAGCTTCA
*PKC*ι	Human	PKCι_F	GCCATCTGCACAGACCGAAT
	Human	PKCι_R	TCCATGGGCATCACTGGTTC
*rela*	Human	RelA_F	GTGGGGACTACGACCTGAATG
	Human	RelA_R	AGATCTTGAGCTCGGCAGTG
*mtor*	Human	mTOR_F	TCGCTGAAGTCACACAGACC
	Human	mTOR_R	CTTTGGCATATGCTCGGCAC
*bcl2*	Human	BCL2_F	AACATCGCCCTGTGGATGAC
	Human	BCL2_R	TTCACTTGTGGCCCAGATAGG
*bax*	Human	BAX_F	ACAGGGGCCCTTTTGCTTC
	Human	BAX_R	CTTGGTGGACGCATCCTGAG
*cytoc*	Human	Cytochorome c_F	AGCGGGAGTGTTCGTTGTG
	Human	Cytochorome c_R	CCTCCCTTTTCAACGGTGTG
*jun*	Human	Jun_F	ACGGCGGTAAAGACCAGAAG
	Human	Jun_R	CCAAGTTCAACAACCGGTGC
*GAPDH*	Human	GAPDH_F	GTCAACGGATTTGGTCGTAT
	Human	GAPDH_R	TGGTGATGGGATTTCCATTG
*RPL27*	Human	RPL27_F	CTGTCGTCAATAAGGATGTCT
	Human	RPL27_R	CTTGTTCTTGCCTGTCTTGT

## Results

### Effect of PKC inhibitor in TRAIL-resistant HT1080 cells

**Figure 1:**
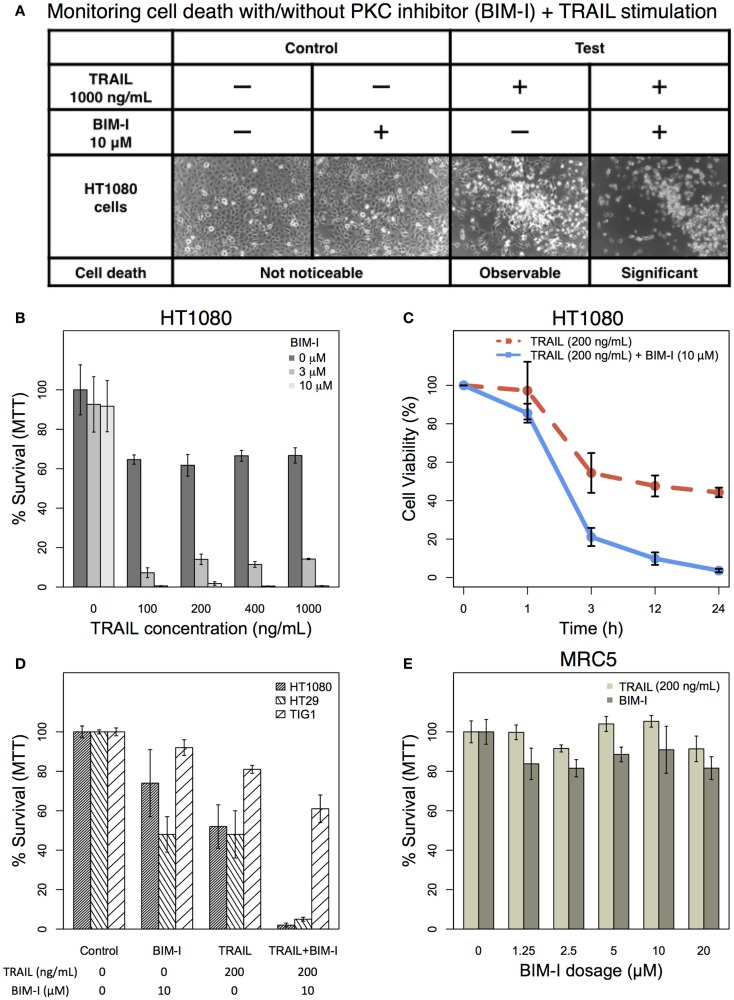
**The effect of TRAIL and PKC inhibitor (BIM-I) on cancer (HT1080 and HT29) and normal (TIG-1 and MRC-5) cells**. **(A)** Phase contrast microscopic images of HT1080 cells in the presence or absence of TRAIL (1000 ng/mL) and/or BIM (10 μM). Living cells appear as adherent cells, while dead cells float in the dish and are highlighted in white. **(B)** TRAIL and BIM-I dosage-dependent cell survival (MTT assay) rates of HT1080 cells (1 × 10^5^), 24 h after treatment (TRAIL: 0, 100, 200, 400, 1000 ng/mL, BIM-I: 0, 3, 10 μM). **(C)** Cell viability (trypan blue assay) of HT1080 cells (3 × 10^5^) at 0 h (no stimulation) and at 1, 3, 12, 24 h after treatment (TRAIL: 200 ng/mL, BIM-I: 10 μM). **(D)** Cell survival (MTT assay) rates of HT1080 (1 × 10^5^), HT29 (1.5 × 10^5^) cancer cells, and TIG-1 (2 × 10^5^) normal cells were observed 24 h after treatment in the presence of TRAIL (200 ng/mL) or BIM-I (10 μM), or both, compared to unstimulated cells (control). **(E)** BIM-I dosage-dependent (0, 1.25, 2.5, 5, 10, 20 μM) cell survival rates of MRC-5 (0.5 × 10^5^) normal cells after TRAIL stimulation (200 ng/mL) were obtained through MTT assay after 24 h. Average cell viability is shown in percentage for *n* = 3 independent experiments. Error bars indicate mean values ± SD.

Based on our previous computational TRAIL model, the removal of PKC family members would enhance HT1080 cell death by 95% (1). Here, we investigated the actual experimental effect of PKC inhibition to HT1080 cells in TRAIL stimulation. HT1080 cells were stimulated with 1000 ng/mL of TRAIL in the presence or absence of 10 μM of PKC inhibitor (31–33), BIM-I, pre-treatment and compared with unstimulated control with and without BIM-I pre-treatment (Figure 1A). We observed, phenotypically, that HT1080 cell death was significantly increased in combinatorial treatment of TRAIL and BIM-I (Figure 1A, forth column), while control pre-treated with BIM-I did not induce any noticeable cell death (Figure 1A, second column).

Next, we investigated cell survival ratio using MTT assays for HT1080 cells pre-treated with BIM-I with increasing dosage (0, 3, and 10 μM) for 30 min prior to increasing TRAIL stimulation (0, 100, 200, 400, and 1000 ng/mL) for 24 h (Figure 1B). Notably, from these experiments, it is clear that HT1080 cell death is almost unaffected with any dosage of BIM-I without TRAIL stimulation. However, when BIM-I was treated in the presence of TRAIL, the effect synergistically produced significant cell death, compared with TRAIL alone (Figure 1B). Remarkably, as predicted by our previous computational TRAIL model (1), the inhibition of PKC (with 10 μM of BIM-I) resulted in about 99% cell death for TRAIL stimulation (with 100 ng/mL or more) in HT1080 cells. We further investigated the cell viability of HT1080 with respect to stimulation time, and noticed that significant cell death occurs at 3 h and onward (Figure 1C).

Next, in addition to HT1080, we also investigated another TRAIL-resistant cancer cell type (HT29) and compared with normal fibroblasts (TIG-1 and MRC-5). Experiment-matched MTT assays revealed that both HT1080 and HT29 cell cultures treated with BIM-I were sensitized to TRAIL-induced cell death (approximately 99 and 95% cell death, respectively), while normal TIG-1 and MRC-5 largely survived (Figures 1D,E). These results indicate that PKC inhibitor, BIM-I, has specific ability to enhance cell death in TRAIL-resistant cancer cells while having little effect on normal cells.

### Treatment of PKC inhibitor with TRAIL enhances cell death through apoptosis

**Figure 2:**
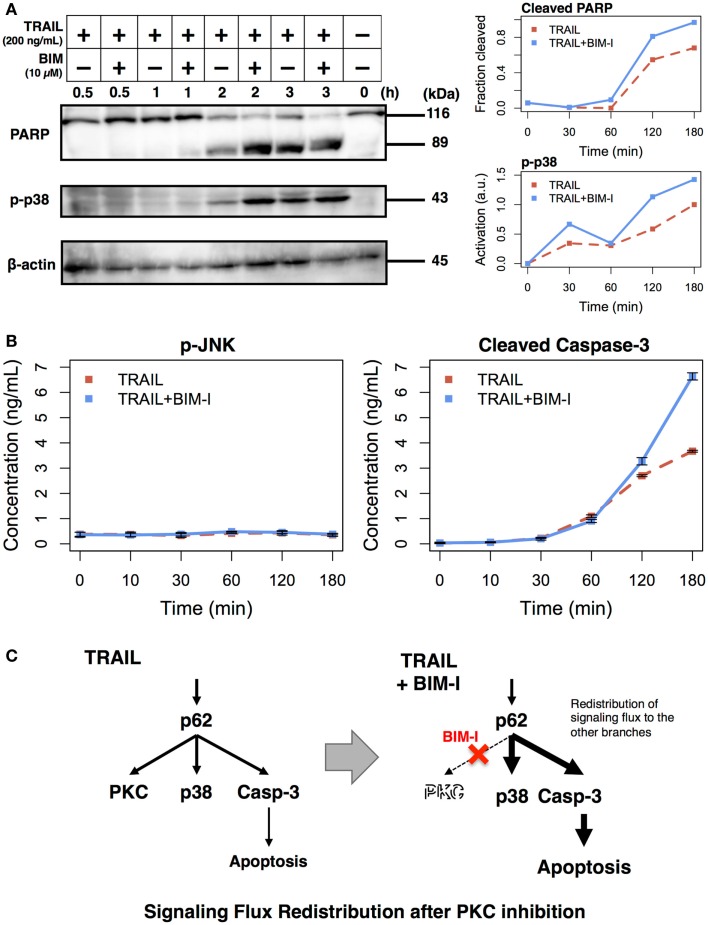
**Enhancement of apoptotic signaling molecules in the presence of BIM-I in TRAIL-stimulated HT1080 cells**. **(A)** Cleavage of PARP, phosphorylation of p38, and concentration of β-actin were determined by western blotting at 0, 30, 60, 120, and 180 min after TRAIL stimulation (200 ng/mL) of HT1080 cells in absence or presence of BIM-I (10 μM). Right panels represent the quantification of fraction of cleaved PARP (top, cleaved PARP/total PARP for each time point) and p38 activation (bottom, relative to maximum value of TRAIL stimulation without BIM-I) using ImageJ (http://imagej.net). **(B)** Phosphorylation of JNK and levels of cleaved caspase-3 protein were measured by ELISA at 0, 10, 30, 60, 120, and 180 min after TRAIL stimulation (200 ng/mL) of HT1080 cells in the absence or presence of BIM-I (10 μM). Error bars indicate mean values ± SD for *n* = 3 independent experiments. **(C)** Schematic representing the mechanism of signaling flux redistribution at p62 pathway junction toward p38 and caspase-3 signaling branches when PKC is inhibited.

The experimental results, so far, are consistent with our previous model simulations. To further scrutinize the result, that is, to explore the origins of cell death, we performed analysis to observe intracellular markers prior to cell death. According to our model, PKC inhibition causes enhancement of apoptotic pathways through *signaling flux redistribution* (SFR) (1, 22). To check whether apoptosis is increased in TRAIL stimulated and BIM-I treated HT1080 cells, we measured PARP cleavage and p38 phosphorylation using western blotting assays, and caspase-3 activation and JNK phosphorylation using enzyme linked immunosorbent assays (ELISAs) (Figure 2).

Consistent with the prediction of computational model, we observed substantial induction of PARP and caspase-3 cleavage, indicating increased apoptosis in HT1080 cells treated with BIM-I when compared with untreated cells in TRAIL stimulation (Figure 2A, top panel and Figure 2B, right panel). We further noticed enhanced p38 activation and low activity of JNK in TRAIL-stimulated cells treated with BIM-I (Figure 2A, middle panel and Figure 2B, left panel), in agreement with our model predictions for SFR at p62 pathway junction (1) (Figure 2C). Note that the housekeeping protein β-actin remained almost unaffected in the western blots. These results clearly demonstrate that BIM-I is a potential therapeutic target for HT1080 treatment.

**Figure 3:**
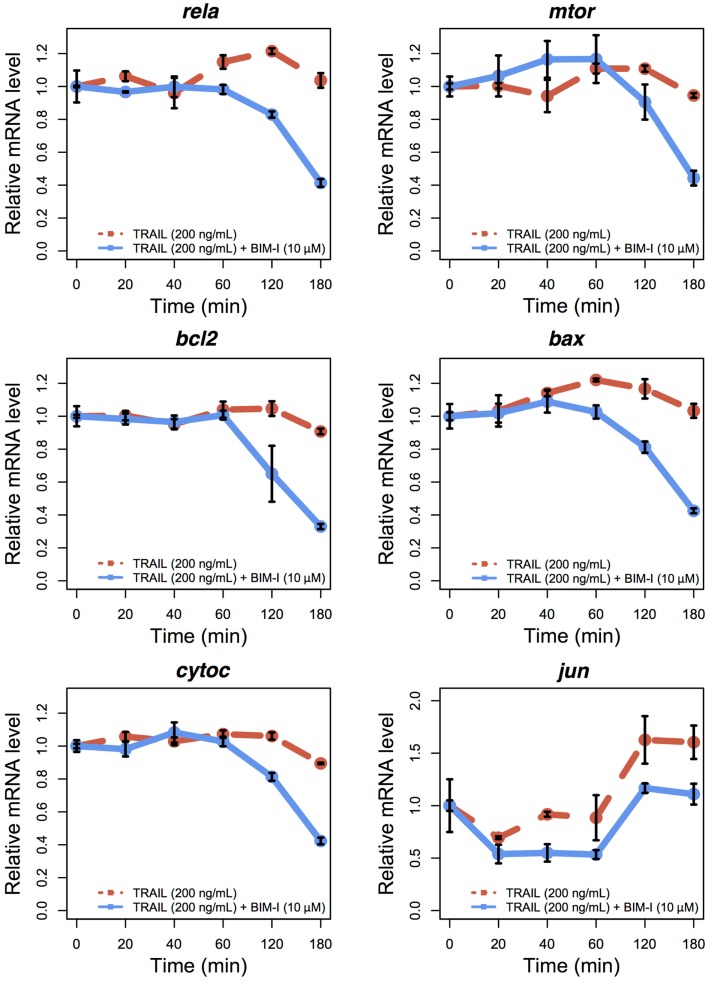
**Temporal relative mRNA expression in TRAIL and BIM-I treated HT1080 cells**. Temporal expression profiles of anti-apoptotic (*rela*, *mtor*, *bcl2*, and *jun*) and pro-apoptotic (*bax* and *cytoc*) genes in HT1080 cells at 0, 20, 40, 60, 120, and 180 min after TRAIL stimulation (200 ng/mL) without (red line) or with (blue line) pre-treatment of BIM-I (10 μM) 30 min prior to TRAIL stimulation. Note that *jun* can also be considered as a pro-apoptotic gene (40). Reported values are the mean expression values (*n* = 3 independent experiments) relative to time 0 of each condition. Error bars indicate mean values ± SD.

To examine the expression levels of appropriate genes in TRAIL-stimulated HT1080, with and without BIM-I, we performed qRT-PCR experiments for several survival and apoptotic genes (*rela*, *mtor*, *bcl2*, *bax*, *cytoc*, and *jun*) at 0, 20, 40, 60, 120, and 180 min (Figure 3). Except for *jun*, the levels of genes were stable for up to 60 min, after which their expressions were significantly reduced, especially for BIM-I treated HT1080 cells, in correlation with the cell death dynamics (Figure 1C). These data indicate that, except for *jun*, transcription of the genes does not occur, perhaps due to the increased signaling flux through the apoptosis process depriving transcriptional signaling and, or due to the repression of pre- and post-transcriptional mechanisms found during apoptosis (34–38). Our observations are also consistent with other TRAIL-induced apoptosis studies investigating gene expressions in HeLa (35) and MCF7 (36) cells.

Interestingly, *jun* levels showed an initial decrease during the first 20 min and then increased and stabilized after 120 min. This pattern indicates *jun* may evade the global transcriptional repression and play a role during apoptosis. Such behavior has been previously observed for other genes, in particular, genes translated through internal ribosome entry site (IRES)-mediated translation, which is known to occur during apoptosis after TRAIL stimulation of MCF7 cells (36, 39). Notably, the presence of IRESs in *jun* transcriptional machinery has also been previously shown (40). Nevertheless, further investigation is required to define the exact role of *jun* during TRAIL and BIM-I mediated apoptosis.

Overall, the experiments demonstrate that the enhancement of cell death of BIM-I pre-treated TRAIL-stimulated cancer occurs through apoptosis.

### Identification of specific PKC isoform target for enhanced cell death

Although we have demonstrated that PKC is a key target to enhance apoptosis in TRAIL-resistant cancer cells, it is unknown which PKC family isoform, among the 10 major members (α, β, γ, δ, ε, ι, θ, η, ζ, and μ), is a crucial single target. To investigate this, we first measured the mRNA expressions of all 10 isoforms (the sequence of primers are available in Table 1) in unstimulated HT1080 cells using qRT-PCR.

**Figure 4:**
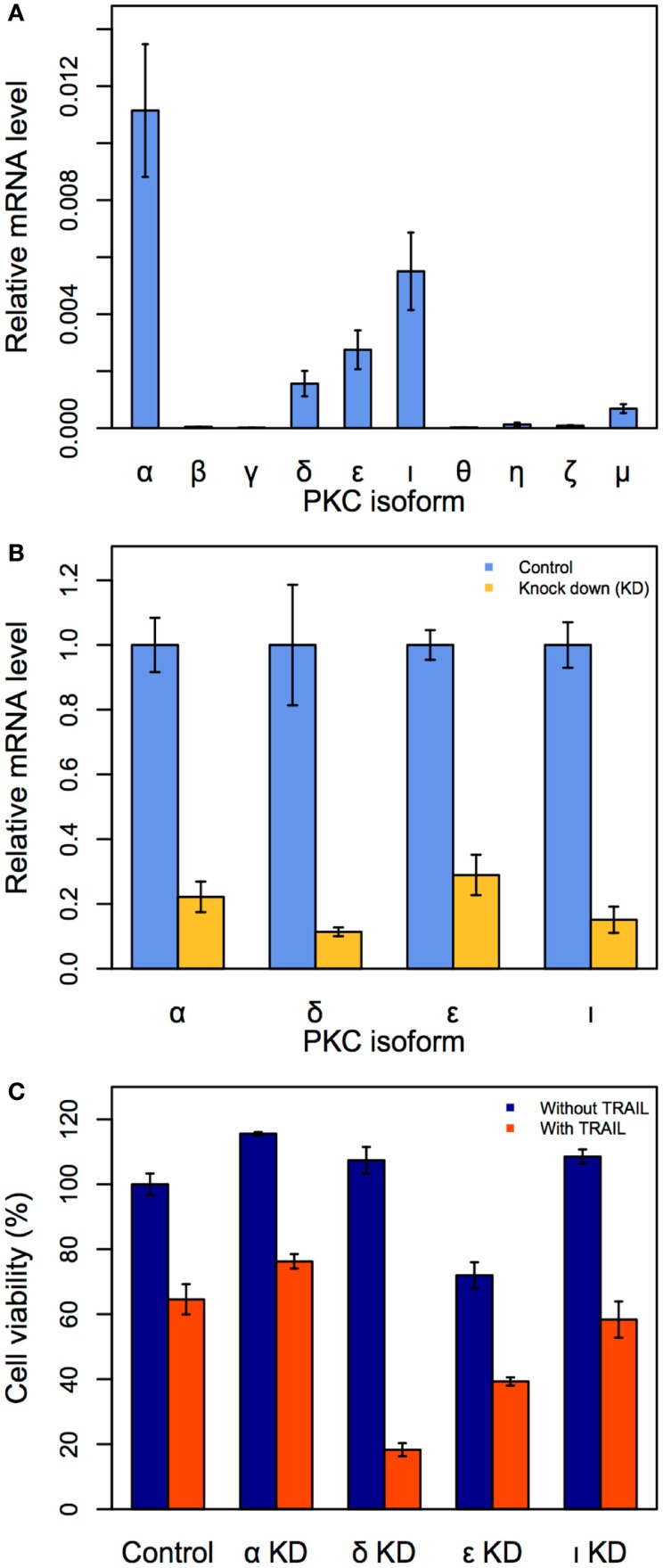
**Identification of the specific PKC isoform target to enhance apoptosis in HT1080 cells**. **(A)** Relative mRNA expressions of 10 PKC isoforms in HT1080 unstimulated cells. **(B)** Effect of siRNA knock-down (KD) for *PKC*α, *PKC*ι, *PKC*ε, and *PKC*δ. HT1080 cells were incubated in the presence of each PKC isoform siRNA (50 μM) for 24 h. Relative mRNA expressions of four PKC isoforms are measured by qRT-PCR. **(C)** Cell viability assay (trypan blue) of HT1080 cells incubated in the presence of PKC isoforms siRNA (50 μM) for 3 h. Error bars indicate mean values ± SD. for *n* = 3 independent experiments.

We observed the gene expressions of four PKC isoforms (α, δ, ε, and ι) were noticeably elevated, indicating that these isoforms may be crucial targets (Figure 4A). To investigate the effect of suppressing each of the four isoforms in TRAIL-stimulated HT1080 cells, we next performed siRNA-mediated PKC (α, δ, ε, and ι) KDs. The effect of each PKC KD was first confirmed after 24 h (Figure 4B). Consequently, we investigated cell viability by trypan blue for each of the four PKC KD conditions with and without TRAIL stimulation (200 ng/mL). Notably, PKCδ KD produced the most significant cell death of approximately 83% after 3 h (Figure 4C). Note that this result is almost identical to TRAIL-stimulated HT1080 pre-treated with BIM-I at 3 h (Figure 1C). Thus, our experiments reveal that PKCδ is the optimal single target for enhancing cancer apoptosis in TRAIL-based therapy.

## Discussion

TRAIL, a proinflammatory cytokine produced by the mammalian immune system, is known to induce apoptosis in cancer cells while leaving non-diseased cells largely unharmed (41, 42). Hence, there has been intense interest in using TRAIL has a therapeutic target to treat cancers (43, 44). However, not all cancers respond to TRAIL (45, 46).

Previously, we investigated the TRAIL-resistant mechanism in HT1080 cells using a computational model (1). We predicted that the suppression of a novel pro-survival molecule would result in significant enhancement of apoptosis through *signaling flux redistribution* (22). PPI database search indicated that the pro-survival molecule is a member of PKC. To experimentally validate this result, in this article, we investigated the effects of two TRAIL-resistant cancer cells to PKC inhibition.

First, using different doses of PKC inhibitor BIM-I together with various levels of TRAIL stimulation, we observed approximately 99 and 95% cell death occurred for HT1080 and HT29 cells, respectively (Figure 1). Notably, the effect on control TIG-1 and MRC-5 cells were less significant, at approximately 40 and 20% cell death, respectively.

Second, to confirm the mechanism for cell death is through apoptosis, we measured the activations of PARP and caspase-3 over 3 h in TRAIL-stimulated HT1080 cells untreated and treated with BIM-I, and compared with activations of p38 and JNK. We found that PARP, caspase-3 cleavages and p38 phosphorylation were significantly enhanced in BIM-I treated cells (Figure 2), while JNK activity was very low. These results are in consistency with the previous prediction of our computational model (1). We also investigated the expressions of major pro- and anti-apoptotic genes, and found them to be mostly repressed at their transcription levels, especially after 1 h for BIM-I treated cells (Figure 3).

Third, to identify the crucial PKC family member for single specific target, we investigated the mRNA expressions of all 10 major isoforms in HT1080 cells. We selected the top four significantly expressed isoforms for developing siRNA KDs, and subsequent experiments demonstrated that PKCδ is a key target for enhancing cell death in TRAIL-resistant HT1080 cells (Figure 4).

It is worthy to mention other previous works that have studied PKC in different cancer types (47–50). Although these works have demonstrated the importance of PKC, the investigations were performed in different cell lines or stimulations. In this work, however, we focused mainly on HT1080 and limitedly on HT29 cells. In addition, we bring to the attention the power of using multidisciplinary research to systemically identify a key target that can be experimentally tested. Therefore, to our knowledge, this is the first time the usefulness of a computational model is shown to identify a consistent and key target for regulating TRAIL-resistance. In summary, our work provides further evidence for the utility of systemic approaches in providing effective treatment strategies to tackle complex diseases.

## Author Contributions

Kentaro Hayashi and Kumar Selvarajoo conceptualized and designed the study. Kentaro Hayashi and Sho Tabata performed the wet lab experiments. Masaru Tomita and Kumar Selvarajoo provided cells, reagents, and discussions. Kentaro Hayashi, Vincent Piras, and Kumar Selvarajoo wrote the article. All authors read and approved the final manuscript.

## Conflict of Interest Statement

The authors declare that the research was conducted in the absence of any commercial or financial relationships that could be construed as a potential conflict of interest.
